# Comparison of Leaf Proteomes of Cassava (*Manihot esculenta* Crantz) Cultivar NZ199 Diploid and Autotetraploid Genotypes

**DOI:** 10.1371/journal.pone.0085991

**Published:** 2014-04-11

**Authors:** Feifei An, Jie Fan, Jun Li, Qing X. Li, Kaimian Li, Wenli Zhu, Feng Wen, Luiz J. C. B. Carvalho, Songbi Chen

**Affiliations:** 1 Tropical Crops Genetic Resources Institute, Chinese Academy of Tropical Agricultural Sciences/Key Laboratory of Ministry of Agriculture for Germplasm Resources Conservation and Utilization of Cassava, Hainan, China; 2 Analysis and Testing Center, Jiangsu University, Jiangsu, China; 3 Proteomics Core Facility, Department of Molecular Biosciences and Bioengineering, University of Hawaii at Manoa, Manoa, Hawaii, United States of America; 4 Guangxi Sub-tropical Crop Research Institute, Nanning, China; 5 Genetic Resources and Biotechnology, Embrapa, Brazil; University of South Florida College of Medicine, United States of America

## Abstract

Cassava polyploid breeding has drastically improved our knowledge on increasing root yield and its significant tolerance to stresses. In polyploid cassava plants, increases in DNA content highly affect cell volumes and anatomical structures. However, the mechanism of this effect is poorly understood. The purpose of the present study was to compare and validate the changes between cassava cultivar NZ199 diploid and autotetraploid at proteomic levels. The results showed that leaf proteome of cassava cultivar NZ199 diploid was clearly differentiated from its autotetraploid genotype using 2-DE combined MS technique. Sixty-five differential protein spots were seen in 2-DE image of autotetraploid genotype in comparison with that of diploid. Fifty-two proteins were identified by MALDI-TOF-MS/MS, of which 47 were up-regulated and 5 were down-regulated in autotetraploid genotype compared with diploid genotype. The classified functions of 32 up-regulated proteins were associated with photosynthesis, defense system, hydrocyanic acid (HCN) metabolism, protein biosynthesis, chaperones, amino acid metabolism and signal transduction. The remarkable variation in photosynthetic activity, HCN content and resistance to salt stress between diploid and autotetraploid genotypes is closely linked with expression levels of proteomic profiles. The analysis of protein interaction networks indicated there are direct interactions between the 15 up-regulation proteins involved in the pathways described above. This work provides an insight into understanding the protein regulation mechanism of cassava polyploid genotype, and gives a clue to improve cassava polyploidy breeding in increasing photosynthesis and resistance efficiencies.

## Introduction

Cassava (*Manihot esculenta* Crantz) is a perennial shrub of the *Euphorbiaceae* family. It is a major calorie source for sub-Saharan Africans and is ranked the sixth most important source of calorie in human diet worldwide [1]. In south China, cassava mainly provides raw materials to produce starch and biofuel. It is a potential crop to grow in semi-arid lands if a suitable cultivar is bred [2]. However, cassava breeding faces several limitations such as (1) its heterozygous genetic makeup which makes it time consuming to breed efficiently and (2) low tolerance to salinity and cold which makes it difficult to grow in north China and saline soils [3]. Because of its importance, a number of important studies regarding genome, proteome and transcriptome have been performed. A draft genome sequence and limited proteome identification have been generated to provide new clues for cassava breeders to overcome the limitations [3]–[6].

One of the potential approaches to increase stress resistance is to produce polyploidy (whole-genome duplication) [2]. The polyploid crops, influenced by nuclear genome size, have much larger cells than the diploid ones [7]. Several studies showed that the significance of the proportional increase in cell volume with increase in DNA content [8], [9]. This cell volume increase along with DNA content increase was observed in secondary xylem radial and vasicentric axial parenchyma cells, and radial parenchyma and sieve elements of secondary phloem [2], [8], [9]. Additionally, DNA content increase in the synthesized polyploids led to the description of structural changes such as chromosomal rearrangements and gains or losses of DNA sequences, demonstrating the occurrence of modifications at the level of gene expression. The studies from Brassica, potato and cotton polyploids have shown that some genes are silenced after polyploidization, while others are derepressed [10]–[12], which suggests that functional and phenotypic evolution may be driven by these genomic changes.

Although these have been significant recent advances on the genomic and transcriptomic consequences of genomic merger and doubling, the fate of translated gene products, i.e., the proteome, remains poorly studied in the context of polyploidization [12], [13]. Using two-dimensional electrophoresis (2-DE) combined mass spectrometry (MS), it is often possible to visualize, quantify, and identify hundreds or even thousands of proteins in a given tissue or cell sample. Proteome analysis is increasingly used in functional plant studies. Proteomic analysis has the potential to provide a broad view of plant responses to stress at the level of proteins [14], [15]. Some reports have been focused on proteomic analysis of plant polyploids including cabbage [16], wheat [17]–[19], cotton [12], potato [20], banana [21], *Arabidopsis* [22] and *Tragopogon* [23]. However, little is known about the effect of polyploidy on the cassava global protein networks that underlie key physiological or developmental processes.

In the present study we investigate and validate the changes of proteome patterns between cassava NZ199 polyploid genotype and its diploid plants using 2-DE combined MALDI-TOF-MS/MS. All differential proteins were clustered into cohesive groups based on their biochemical functions. The biological network of protein-protein interaction was established to describe the polyploid photosynthetic activity tolerance against stresses. Our data will provide crop breeders a set of protein database involved in cassava polyploidy and would be useful to understand the polyploidy mechanism involved in the key physiological or developmental processes.

## Materials and Methods

### Analysis of different ploidy levels in cassava

Cassava cultivar NZ199 autotetraploid genotype was ployploidized artificially by the use of colchicine applied as a solution of 0.001% to lateral buds over a period of 72 h. The emerging shoots were screened for the formation of chimeras or total tetraploids with the chimeras eliminated. To identify autotetraploids, buds were observed for standard chromosome counting and flow cytometric analysis (CellLab QuantaTM SC MPL, Beckman, USA) [24] in addition to observing leaf shape. The shoots were propagated vegetatively and grown at the Cassava Germplasm Pool, Tropical Crops Genetic Resources Institute, Chinese Academy of Tropical Agricultural Sciences. Functional leaves on the top of cassava diploid and autotetraploid plants grown for 180 d at CGB were sampled for protein extraction. One leaf was collected from one cassava plant, and three leaves were collected.

### Protein extraction and 2-DE separation

Collected leaves were washed with distilled water and the central nervure was removed. Leaves were cut into pieces and 0.5 g pieces were weighed for protein extraction. Leaf proteins were extracted with phenol according to the procedure of Chen et al. [25]. Protein pellets were dissolved in sample buffer [9.5 M urea, 2 M Thiourea, 4% (w/v) CHAPS, 1% (w/v) DTT, 2.5 mM EDTA, 2.5 mM EGTA], and the protein content was then measured with the protein assay according to the instruction manual (Bicinchoninic acid protein assay kit, product code BCA-1 and B9643, Sigma). For analytical and preparative gels, the 13 cm IPG strips (pH 4–7, linear) (GE Healthcare Bio-Sciences AB) were rehydrated overnight with 312 µl of rehydration stock solution [7 M urea, 2 M thiourea, 3% (w/v) CHAPS, 0.5% (v/v) Triton X-100, 1% (v/v) IPG buffer, and 0.002% (w/v) bromophenol blue], containing 300 µg of protein, at room temperature. Isoelectric focusing (IEF) was conducted at 20°C with a Multiphor II (Amersham Biosciences). The running conditions were as follows: 300 V for 0.05 h, then, increased from 300 V to 3500 V as a gradient over 1.5 h, and finally 3500 V for 4.20 h. The focused strips were equilibrated twice for 15 min each first in 1% (w/v) DTT and then 2.5% (w/v) iodoacetamide prepared in equilibration buffer (50 mM Tris-HCl (pH 8.8), 6 M urea, 30% (v/v) glycerol, 2% (w/v) SDS). The second-dimension electrophoresis was performed by SDS-PAGE in a vertical slab of 12% acrylamide using an SE 600 Series Vertical Slab Gel Unit (Hoefer Scientific Instruments, San Francisco, CA, USA). Preparative gels were stained with Colloidal Coomassie brilliant blue G-250 [26]. Three independent biological replications were carried out.

### Image and data analysis

Gel matching for protein quantification was performed by Image Scanner III (GE healthcare) and Delta 2D (DECODON GmbH, Greifswald, Germany) software, and spot pairs were confirmed visually. The differentially expressed spots were determined by using Scheffe's test at P<0.05. The abundance of each protein spot was estimated by the percentage volume (% Vol). Only those with significant and reproducible changes were considered to be differentially accumulated proteins.

### Tryptic in-gel digestion

Briefly, the differential spots were excised from 2-DE gels and cut into ∼1 mm^3^, and then washed twice in MillQ water for 10 min. The washed gel pieces were subjected to destaining solution (25 mM ammonium biocarbonate, 50% acetonitrile) followed by sonication for 5 min. The gel pieces were again washed twice in MillQ water for 10 min, followed by washing twice in 50% acetonitrile for 10 min. After dehydration with 50 µl acetonitrile for 2–3 min, the gel pieces were digested overnight at 37°C in 15 µl of sequencing grade trypsin (Promega) according to the manufacturer's instructions (1 µg in 100 µl of 25 mM ammonium biocarbonate). The supernatants were transferred to a fresh tube and stored at 4°C until analysis.

### Protein identification by MALDI-TOF-MS/MS

Differential proteins were identified using MALDI-TOF-MS/MS at Analysis and Testing Center, Jiangsu University. After 1 µl peptide extract produced by the in-gel digestion was placed on an anchor chip and air-dried, it was covered with 1 µl solution of 0.4 mg/ml α-cyano-4-hydroxycinnamic acid in a mixture of acetonitrile and 0.1% trifluoroacetic acid (TFA) (70∶30) and then air-dried. The mass spectra were acquired on an Ultraflex-TOF mass spectrometer (Bruker, German). Spectra were internally calibrated with trypsin auto-digestion products. Data captured by MALDI-TOF-MS/MS were matched via Mascot v2.2.03 (http://www.matrixscience.com) against NCBI [Taxonomy: Viridiplantae (Green Plants)]. Carbamidomethyl (Cys) and oxidation (Met) were considered as variable modifications. A single missed cleavage was permitted. Peptide mass tolerance was set as 3.0 Da and MS/MS ion mass tolerance was set at 1.5 Da. Peptide charge states (+1, +2, +3) were taken into account. Routine protein identification required sequence-confirmed data for a minimum of one peptide with recognition as the top ranking match in the Mascot Standard scoring system [3].

### Western blot analyses

Leaves of cassava cultivar NZ199 diploid and autotetraploid genotypes were homogenized. The protein extraction and Western blot were performed according to the method previously reported [3]. Proteins detected by immuno-staining with antiRubisco-polyclonal antibody (AS07218), anti-APX antibody (AS08368) and anti-PrxQ antibody (AS05093) from Agrisera. Western blots were developed according to the method of NBT/BCIP from Roche (11681451001).

### Photosynthetic activities measurement by imaging pulse amplitude modulation

The Maxi-version of the Imaging Pulse Amplitude Modulation (Imaging PAM) and the software Imaging WIN version 2.39 (both Heinz Walz GmbH, Effeltrich, Germany) were used to determine the photosynthetic activities according to Behr et al. [27]. The selected leaves attached to cassava cultivar NZ199 diploid and autotetraploid genotypes were adjusted in the dark for 20 min prior to measurement. The leaves were detached at 10:00 a.m (ambient full sunlight). The detached leaf was clamped onto the holder at a distance of 7 cm between the leaf and the CCD camera. The other variables were as follows: light intensity at 2, frequency at 1, gain at 7, damping at 2; saturation pulse with intensity at 10 and width at 8. Each recording started by determining the dark fluorescence parameter F_0_. A saturating light pulse was employed to determine the maximal fluorescence parameter Fm. The maximal quantum yield of PS II was calculated as Fv/Fm = (Fm-F_0_)/Fm. In the presence of light intensity 185 µE m^−2^ s^−1^, the current fluorescence yield (F_t_) and the maximum light adapted fluorescence (Fm′) were measured to calculate the effective PS II quantum yield (ΦPSII = (Fm′-F_t_)/Fm′). Additionally, the nonphotochemical quenching [NPQ = (Fm-Fm′)/Fm] was determined to show the proportion of absorbed light energy that is not used for photosynthetic electron transport. NPQ/4 is NPQ divided by 4 in order to allow for a display in a color code ranging from 0.0 to 100%. For quantitative analyses of the whole leaves, the software used 5 areas of interest of 25 mm^2^ that were randomly distributed over each leaf segment measured. For each variant, three individual plants were used and the respective results were averaged [27].

### Salt-stress detection

The stems (length about 1.0–1.5 cm with one axillary bud) of cassava NZ199 diploid and autotetraploid genotypes, obtained from *in vitro* tissue culture at CGB, were cultured on MS [0.03 mg l^−1^ naphthylacetic acid (NAA), 3% sucrose, 0.3% Gelrite, pH5.8–6.0] and salt stress medium (50 mM NaCl, 0.03 mg l^−1^ NAA, 3% sucrose, 0.3% Gelrite, pH5.8–6.0), respectively. Three stems were cultured in one tissue-culture bottle and incubated in the tissue culture room at 26–28°C under a 12-h photoperiod. The length and fresh weight of 12 plantlets of both genotypes in salt-stressed medium were measured after 50 days.

### Determination of cyanogenic glucosides (CGs) by HPLC

Five plantlets, grown for 2 months *in vitro*, were selected from each cassava cultivar NZ199 diploid and autotetraploid genotypes. A leaf disc was sampled from the first unfolded leaf of each plant by snap-closing a 2 ml-Eppendorf lid around one of the leaf fingers. Five replicates were carried out in each diploid and autotetraploid genotypes. To determine the content of CGs directly, the leaves samples were immersed into 300 µl pre-warmed 85% (v/v) methanol and boiled at once in a water bath at 100°C for 3 min, then, cooled on ice. The methanol extract was transferred to a new tube and filtered through a 0.45 µm filter [28].

CG content was determined on a Hitachi LC 2130 series coupled with autosampler L-2200, UV detector LC 2030 and chromatography workstation^T^ 2100P (Hitachi, Japan). The HPLC column was a Kromasil 100-5C18 column (250×4.6 mm, 5 µm). The mobile phases consisted of water (A) and acetonitrile (B). The flow rate was 0.8 ml/min. The gradient program was as follows: 0 to 30 min, linear gradient 10% to 100% (v/v) B, 30 to 33 min, linear gradient 100% to 10% B, 33 to 38 min. The UV detection wavelength was set at 215 nm. The column temperature was 30°C. The injected volume of samples was 20 µl. The concentration of hydrogen cyanide (HCN) standard sample is 0.5 ppm collected from the National Institute of Metrology of China. The retention time for CG was 28.6 min.

### Generation of protein interaction networks

All proteins identified in cassava cultivar NZ199 autotetraploid were compared with those in diploid genotype and were used to generate a wider protein interaction map by employing a Pathway Studio software program (www.ariadnegenomics.com) [25].

## Results

### Determination of cassava polyploid genotypes

**Figure 1:**
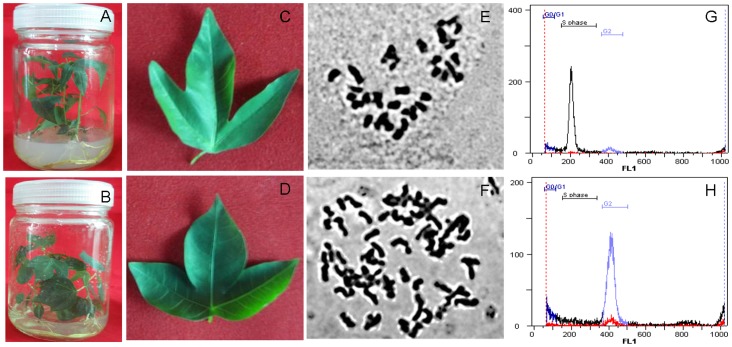
Standard chromosome counting and flow cytometric analysis of different ploidy levels in cassava cultivar NZ199 leaves. A, *in vitro* plantlets of cassava diploid genotype; B, *in vitro* plantlets of cassava autotetraploid genotype; C, leaf of cassava diploid genotype; D, leaf of cassava autotetraploid genotype; E, chromosome number of diploid genotype (2n = 2x = 36); F, chromosome number of autotetraploid genotype (4n = 4x = 72); G, a fluorescence peak of diploid nuclei located at channel position 200; H, a fluorescence peak of autotetraploid nuclei located at channel position 400.

The autotetraploid status was validated under light microscope. Examination using the leaves of confirmed diploid (Fig. 1A and C) and autotetraploid (Fig. 1B and D) cassava NZ199 plants showed that the chromosome number from diploid genotype was 36 (2n = 2x = 36) (Fig. 1E), whereas that of autotetraploid genotype was 72 (4n = 4x = 72) (Fig. 1F). The fluorescence peak of diploid nuclei was located at channel position 200 (Fig. 1G), whereas that of autotetraploid nuclei was located at channel position 400 (Fig. 1H), consistent with the expected DNA relationship of the two types of plants.

### Protein profiles of cassava autotetraploid and diploid genotypes

**Figure 2:**
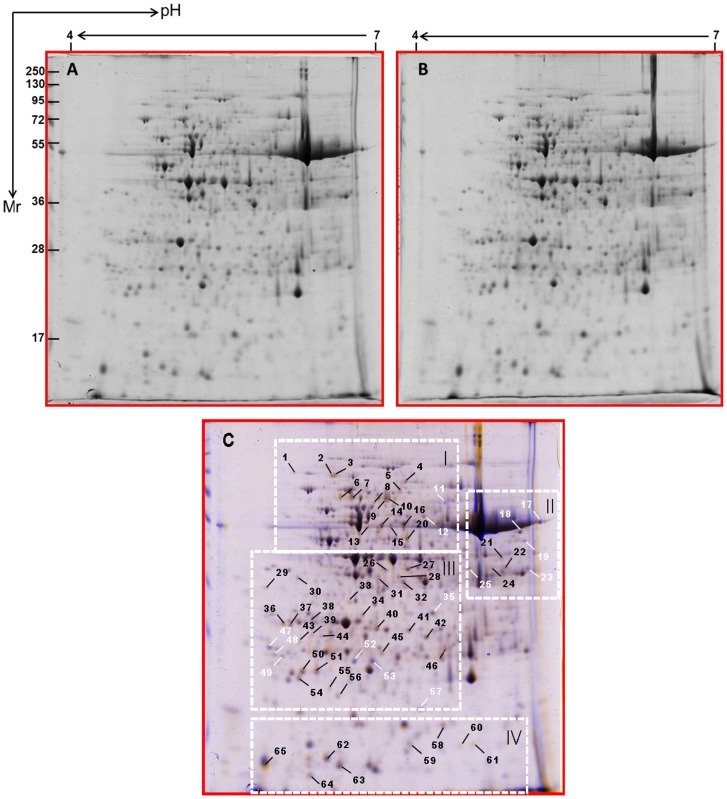
2-D gel protein profiles of leaves from cassava NZ199 diploid (A) and autotetraploid genotypes (B) and wrapped 2-DE map from diploid and autotetraploid genotypes (C). The white and black arrows in pane C indicated proteins that showed detectable changes (>2.0-fold of the normalized volume) in abundance compared with those observed in the control; white indicated a down-regulated match, and black indicated an up-regulated match. Small boxes indicated the gel regions to be amplified to highlight clearly detectable spots in Fig. 3.

**Figure 3:**
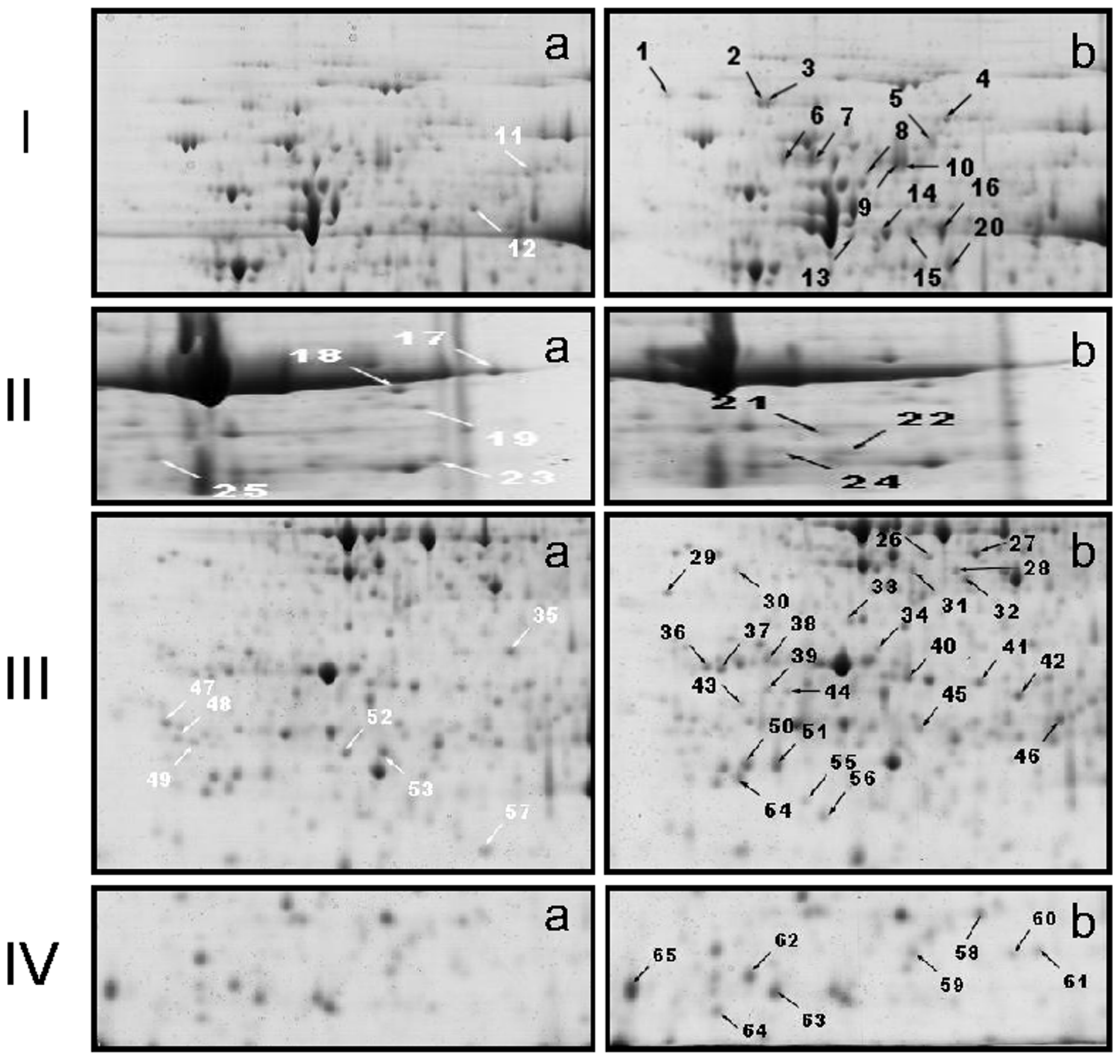
Amplification of small boxes from Fig. 2C to highlight detectable spots that represent differentially abundant expression. In I, II, and III: a, diploid genotype, b; autotetraploid genotype. White arrow indicated a down-regulated match, and black indicated an up-regulated match. The numbers correspond to the 2-DE gel in Fig. 2.

A total of approximately 500 protein spots were detected by digital image analysis, and at least 300 spots gave reproducible staining patterns for all samples as judged by eye and by spot intensity ranking (Fig. 2A and 2B, Figure S1). Using a spot-to-spot comparison and statistical analysis, a total of 65 stained spots (Fig. 2C) from autotetraploid genotype were found to have significant changes (p<0.05) with greater than 2.0-fold altered intensity compared with diploid genotype. The differential spots were determined using Scheffe's test at P<0.05. Of these, the expression of 14 spots was down-regulated and the remainder up-regulated (Fig. 2C). As shown in Fig. 3, the detectable spots with differentially abundant expression in Fig. 2C were clearly highlighted (a, diploid genotype; b, autotetraploid genotype). 3-D images of these spots were generated using Delta2D software to detect the significant changes between diploid and autotetraploid genotypes as shown in Figure S2.

### Functional grouping of identified proteins

**Figure 4:**
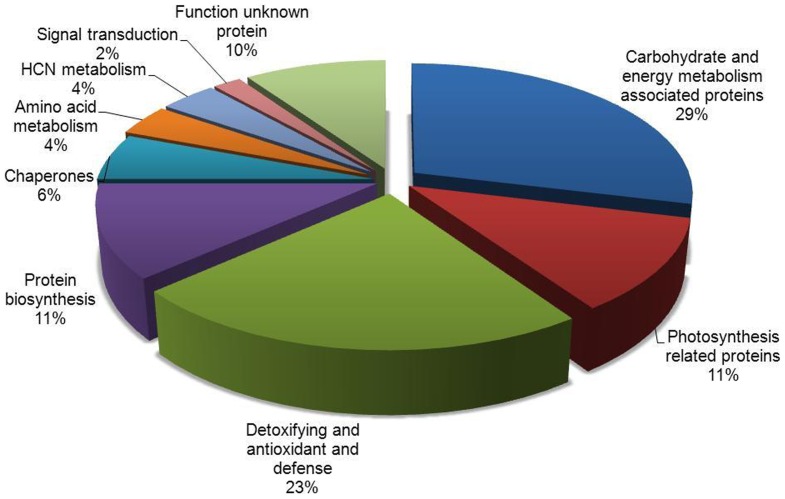
Functional categories of 52 differential proteins identified in cassava NZ199 autotetraploid leaves compared withdiploidgenotypes. Number of spots altered in the expression in the leaves of cassava autotetraploid genotype. Unknown proteins included those whose functions had not been described.

**Figure 5:**
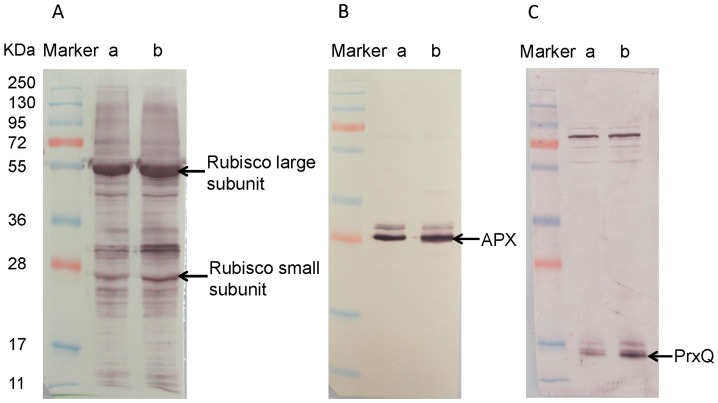
Western blotting of Rubisco, APX and PrxQ. The expression of Rubisco, APX and PrxQ in leaves of cassava NZ199 diploid (a) and autotetraploid (b) genotypes were detected by western blotting using antiRubisco-polyclonal antibody (AS07218), anti-APX antibody (AS08368) and anti-PrxQ antibody (AS05093) from Agrisera, respectively.

Sixty-five spots with differential expression were isolated from 2-DE gels and identification performed using MALDI-TOF-MS/MS, of which fifty-two protein spots were identified. Other 13 spots, however, remained unidentified. Forty-seven identified proteins were up-regulated and 5 were down-regulated in autotetraploid genotype compared with diploid genotype. Functions of 52 differentially expressed proteins were annotated via the survey of gene banks (Table 1, Fig. 4). Fifteen proteins are associated with carbohydrate and energy metabolism, of which 4 proteins (spot 35, spots 47–49) were down-regulated. The 32 up-regulated proteins were associated with photosynthesis (6 spots, 11%), defense system (12 spots, 23%), HCN metabolism (2 spots, 4%), protein biosynthesis (6 spots, 11%), chaperones (3 spots, 6%), amino acid metabolism (2 spots, 4%) and signal transduction (1 spot, 2%). Other 5 were function-unknown proteins, including 1 down-regulated protein. The characteristics of 4 down-regulated proteins, spot 35 and spots 47–49, relate with energy metabolism. Protein spot 35 was identified as NAD(P)-binding Rossmann-fold-containing protein and spots 47–49 were putative ATP-binding proteins (Table 1). To ensure reliability of differential proteins on 2-DE gels, the protein expressions of Rubisco, APX and PrxQ in leaves of cassava diploid and autotetraploid genotypes were detected by immunoblotting to validate the proteomic analysis (Fig. 5). For example, the result from western blot showed that the expression of Rubisco small subunit in autotetraploid genotype was more than that in diploid genotype, which is similar with that seen on 2-DE analysis (Figs. 3 and 5).

**Table 1:** Identification of differential proteins in cassava cultivar NZ199 leaves from autotetraploid and diploid genotypes.

Spot Number^a^	Identification	Fold changes^b^ (Mean±SE)	Accession no^c^	Theoretical pI/Mw(kDa)	Score^d^/No. of Unique peptides matched^e^
*Carbohydrate and energy metabolism associated proteins(15)*					
13	ATP synthase subunit beta, mitochondrial	2.02±0.05(+)	P17614	5.95/59.86	617/4
14	ATP synthase subunit beta, mitochondrial	2.34±0.11(+)	P17614	5.95/59.86	156/2
15	ATP synthase subunit beta, mitochondrial	2.09±0.06(+)	P17614	5.95/59.86	156/2
16	ATP synthase subunit beta, mitochondrial	2.82±0.09(+)	P17614	5.95/59.86	185/2
26	Phosphoglycerate kinase - *Arabidopsis thaliana*	2.27±0.10(+)	AAB60303	4.93/41.91	69/1
28	alcohol dehydrogenase, putative - *Ricinus communis*	4.86±0.11(+)	XP_002525379	8.61/41.58	92/1
30	ATP synthase beta subunit - *Gunnera manicate*	2.26±0.13(+)	ABV65134	5.23/54.10	74/1
35	NAD(P)-binding Rossmann-fold-containing protein - *A. thaliana*	2.06±0.06(−)	NP_565868	8.37/34.88	263/2
43	putative triosephosphate isomerase - *A. thaliana*	2.52±0.14(+)	AAD29799	7.67/33.35	63/1
46	Triose phosphate isomerase, cytosolic - *Oryza sativa subsp.japonica*	3.53±0.11(+)	P48494	5.38/27.06	63/1
47	Putative ATP-binding protein -*Stenotrophomonas maltophilia K279a*	2.08±0.07(−)	CAQ46869	5.87/30.40	88/1
48	Putative ATP-binding protein - *S. maltophilia K279a*	2.02±0.06(−)	CAQ46869	5.87/30.40	88/1
49	Putative ATP-binding protein - *S. maltophilia K279a*	2.12±0.08(−)	CAQ46869	5.87/30.40	88/1
64	ATP synthase CF1 epsilon subunit - *Spinacia oleracea*	2.19±0.12(+)	NP_054942	6.59/14.70	124/1
65	ATP synthase epsilon chain - *Androya decaryi*	2.08±0.05(+)	CAD22407	5.87/14.28	349/4
*Photosynthesis related proteins(6)*					
4	Nuclear encoded precursor to chloroplast protein - *Pisum sativum*	2.37±0.10(+)	AAA33680	6.55/102.71	67/2
8	Rubisco large subunit-binding protein subunit beta, chloroplastic	2.57±0.11(+)	P08927	5.85/62.98	77/1
22	Oxygen evolving enhancer protein 1 precursor - *Bruguiera gymnorhiza*	2.48±0.10(+)	Q9LRC4_9ROSI	35.116/6.48	94/3
31	Putative Rubisco activase protein - *Zantedeschia hybrid cultivar*	4.71±0.10(+)	AAT12492	5.08/27.69	43/1
55	Cytochrome b6-f complex iron-sulfur subunit, chloroplastic	3.71±0.15(+)	P26291	8.63/24.24	170/2
60	Ribulose 1,5-bisphosphate carboxylase small chain precursor - *M. esculenta*	8.36±0.16(+)	AAF06098	8.33/20.41	120/3
*Detoxifying and antioxidant(6)*					
36	Peroxiredoxin - *Phaseolus vulgaris*	4.58±0.06(+)	CAC17803	5.18/28.62	123/1
39	Ascorbate peroxidase APX2 - *M. esculenta*	2.85±0.08(+)	AAX84679	5.31/27.67	169/2
44	Ascorbate peroxidase APX2 - *M. esculenta*	2.26±0.04(+)	AAX84679	5.31/27.67	169/2
45	ascorbate peroxidase APX2 - *M. esculenta*	2.52±0.11(+)	AAX84679	5.31/27.67	484/4
58	Chain A, Prx D - *Populus Tremula*	2.17±0.09(+)	1TP9_A	5.56/17.43	130/2
59	Glutaredoxin	2.06±0.05(+)	O81187	6.05/11.13	63/1
*Defense(6)*					
9	Beta-glucosidase - *M. esculenta*	2.05±0.07(+)	CAA64442	5.80/63.10	71/1
10	Beta-glucosidase - *M. esculenta*	2.11±0.12(+)	CAA64442	5.80/63.10	71/1
24	Chloroplast latex aldolase-like protein - *M. esculenta* (Manioc)	2.28±0.07 (+)	Q5PYQ2_MANES	33.788/6.22	146/3
29	Metacaspase-9-*A. thaliana*	3.43±0.08(+)	AED90710	5.81/35.51	72/1
33	CDSP32 protein - *Solanum tuberosum*	6.13±0.16(+)	CAA71103	8.07/33.46	101/1
61	Thioredoxin-like protein - *A. thaliana*	8.58±0.18(+)	AAF04439	7.62/16.89	64/1
*Protein biosynthesis(6)*					
2	Translation factor - *Medicago truncatula*	3.71±0.10(+)	Q1S825_MEDTR	5.80/94.08	362/2
3	Elongation factor Tu - *M. truncatula*	3.28±0.11(+)	Q1S824_MEDTR	5.91/94.12	108/1
20	Elongation factor Tu, chloroplastic	4.40±0.40(+)	O24310	6.62/53.05	194/2
41	Proteasome subunit alpha type-5	2.40±0.08(+)	Q9M4T8	4.70/25.98	136/2
42	50S ribosomal protein L4, putative - *R. communis*	2.24±0.06(+)	XP_002525600	8.64/31.21	100/1
50	30S ribosomal protein S8, chloroplastic	2.06±0.08(+)	Q2WGF1	11.18/14.52	67/1
*Chaperones(3)*					
7	hsp70 -*Petunia × hybrida*	2.66±0.11(+)	CAA31663	5.06/70.78	42/1
34	SHOOT1 protein - *Glycine max*	2.26±0.10(+)	AAK37555	5.26/40.24	91/1
63	HSP19 class II - *Citrus× paradisi*	2.02±0.07(+)	AAP33012	8.01/11.14	67/1
*Amino acid metabolism(2)*					
27	Glutamate ammonial ligase, cytosolic- *A. thaliana*	2.24±0.10(+)	S18603	5.40/40.73	57/1
56	mitochondrial glycine decarboxylase complex H-protein -*Populus tremuloides*	2.27±0.08(+)	ABO61731	4.78/17.62	168/2
*HCN metabolism(2)*					
6	Linamarase-*M. esculenta*	4.99±0.23(+)	AAB22162	5.52/61.37	260/2
32	acetone-cyanhydrin lyase- *M. esculenta*	2.27±0.08(+)	S45682	6.15/29.50	179/3
*Signal transduction(1)*					
37	14-3-3 protein- *M. esculenta*	4.73±0.06(+)	ADD92154	4.79/29.81	68/1
*Function unknown protein(5)*					
21	predicted protein-*Physcomitrella patens subsp. patens*	2.25±0.06(+)	EDQ53885	6.76/46.38	89/2
38	unnamed protein product -*S. oleracea*	2.21±0.10(+)	CAA29062	5.58/35.04	314/3
40	unnamed protein product -*M. esculenta*	2.38±0.11(+)	CBC70131	5.31/27.67	112/1
52	Predicted protein-*Populus trichocarpa*	3.26±0.16(−)	XP_002325568	9.02/26.95	56/1
54	forkhead-associated domain-containing protein -*Arabidopsis lyrata subsp. lyrata*	2.16±0.06(+)	XP_002878556	8.46/22.23	88/1

The spots showing differential expression (>2.0-fold of the normalized volume) were counted after gel analysis and manual editing with Delta2D software. Each value represents the mean **±** SE of triplicates. Protein spots whose abundance increased (+) or decreased (−) after polyploidy were shown. The numbers corresponded to the 2-DE gel in Fig. 2.

a . The numbers corresponded to the 2-DE gel in Fig. 2–3.

b , Expression change level in tetraploid genotype compared with diploid genotype,

c , NCBI accession number.

d , Probability-based MOWSE (molecular weight search) scores.

e , The number of unique peptides identified by MS/MS, and individual ions scores are all identity or extensive homology (p<0.05).

### Photosynthetic activities in cassava diploid and autotetraploid genotypes

**Figure 6:**
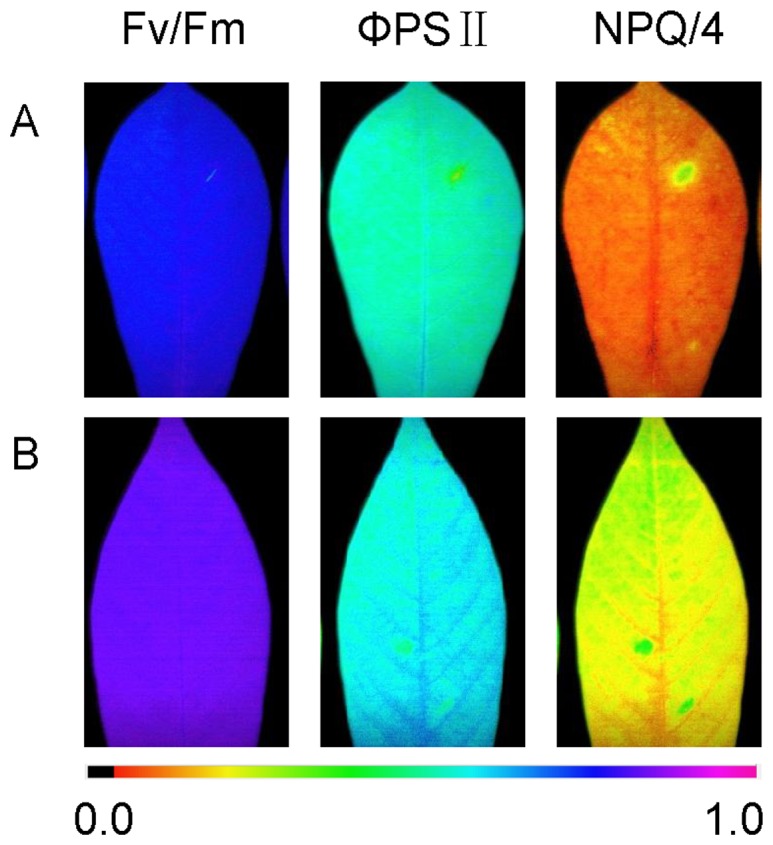
Imaging pulse amplitude modulation of cassava leaves from NZ199 diploid and autotetraploid genotypes. A, diploid genotype; B, autotetraploid genotype; Parameters shown are Fv/Fm [maximal photosystem II (PSII) quantum yield], ΦPSII (effective PSII quantum yield) (at 185 µE m^−2^ s^−1^), and NPQ/4 (nonphotochemical quenching) (at 185 µE m^−2^ s^−1^). The color gradient provides a scale from 0 to 100% for assessing the magnitude of the parameters.

Imaging-PAM studies were performed with the leaves from 50 d-old *in vitro* cassava plants of diploid and autotetraploid genotypes to understand the effects of up-regulated proteins involved in leave photosynthesis metabolism on the photosynthetic activities of cassava polyploid plants. In the present study, we chose the sensitive photosynthesis parameters Fv/Fm, ΦPSII and NPQ/4 to determine the changes of photosynthetic activities. Fig. 6 and Table 2 showed that autotetraploid induction could produce significant effects on the efficiency of excitation energy capture by open Fv/Fm, ΦPSII and NPQ/4, suggesting that an increase in maximal and effective quantum yield and a concomitant increase in NPQ/4 processes are sensitive markers for polyploid genotypes. These data imply up-regulated proteins associated with photosynthesis will result in increase of photosynthetic activities in cassava autotetraploid genotype.

**Table 2:** Photosynthetic parameters collected from cassava cultivar leaves of NZ199 diploid and autotetraploid genotypes.

Cassava genotypes	Fv/Fm (Mean±SE)	ΦPSII (Mean±SE)	NPQ/4 (Mean±SE)
NZ199 diploid	0.753±0.012 A	0.525±0.003 A	0.098±0.012 A
NZ199 autotetraploid	0.828±0.007 B	0.587±0.009 B	0.201±0.019 B

Values were means ± SE. Different capital letters in the same column indicated statistically significant differences according to Duncan test (P<0.01).

### Effects of salt stress on cassava diploid and autotetraploid genotypes

**Figure 7:**
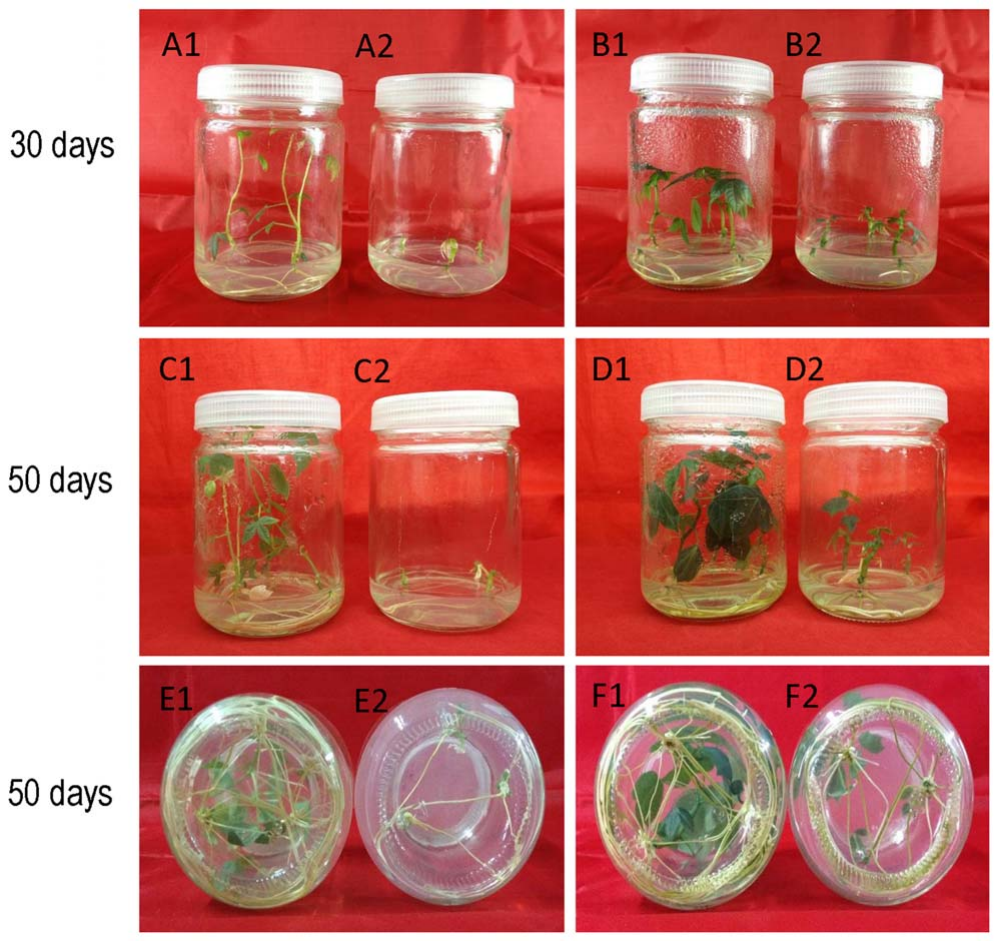
Effects of salt stress on the growth of cassava NZ199 diploid and autotetraploid genotypes. Cassava NZ199 diploid and autotetraploid genotypes were grown at MS medium with 0.03/L NAA used as control, and salt-stressed medium contained MS medium with 0.03 mg/L and 50 mM sodium chloride. A1, C1 and E1 (roots), diploid control; A2, C2 and E2 (roots), salt-stressed diploid plantlets; B1, D1 and F1 (roots), autotetraploid control; B2, D2 and F2 (roots), salt-stressed autotetraploid plantlets.

Plantlets of cassava diploid and autotetraploid genotypes grown on the MS medium and salt-stressed medium for 30 d and 50 d were examined to observe the effects of up-regulated proteins on salt stress tolerance (Fig. 7). The length and fresh weight of 50 d-old cassava plantlets grown on salt-stressed medium showed inhibitory effects of high salt concentrations on the growth of both cassava genotypes. However, length and fresh weight of cassava plantlets from diploid genotype in the presence of salt were significantly lower than those from autotetraploid genotype (Table 3). These data imply association of the up-regulated proteins with the increase of salt stress tolerance.

**Table 3:** The growth of *in vitro* plantlets of cassava NZ199 diploid and autotetraploid genotypes under salt stress.

Salt stress to cassava NZ199 genotypes	Shoot Height (Mean±SE)	Root Length (Mean±SE)	Root Weight (Mean±SE)	Aboveground Weight (Mean±SE)
Diploid control	10.43±0.25 A	10.43±0.21 A	0.16±0.02 B	0.21±0.01 B
Diploid salt stress	1.3±0.10 D	5.53±0.29 C	0.05±0.02 C	0.04±0.01 C
Autotetraploid control	6.83±0.42 B	10.57±0.21 A	0.27±0.03 A	0.42±0.02 A
Autotetraploid salt stress	3.43±0.32 C	7.80±0.53 B	0.17±0.02 B	0.22±0.04 B

Values were means ± SE. Different capital letters in the same column indicated statistically significant differences according to Duncan test (P<0.01).

### Variation in CG content of cassava autotetraploid and diploid genotypes

**Figure 8:**
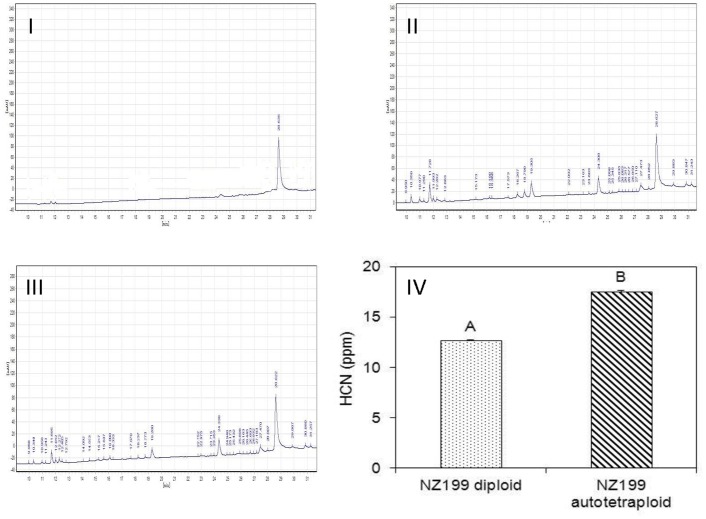
Chromatograms of cyanogenic glucoside of cassava cultivar leaves from NZ199 diploid and autotetraploid genotypes. I, HCN standard sample (0.5 ppm); II, NZ199 diploid genotypes; III, NZ199 autotetraploid genotype; IV, Extraction yield of cyanogenic glucoside from diploid and autotetraploid genotypes. Chromatographic conditions were: Kromasil 100-5C18 column (250×4.6 mm, 5 µm), gradient elution with aqueous acetonitrile, flow rate of 0.8 ml/min, UV detection at 215 nm, and column temperature at 30°C.

Variations of CG content in the leaves collected from 50 d-old *in vitro* cassava plants of diploid and autotetraploid genotypes were studied to validate the effects of up-regulated proteins in relevance with HCN metabolism (spots 6 & 32) in autotetraploid genotype. The CG content of autotetraploid genotypes significantly increased in comparison to diploid genotypes (Fig. 8I–IV). These data imply that an increased conversion of HCN from cyanide containing compounds may be related with up-regulated proteins (linamarase and acetone-cyanhydrin lyase) which are involved in HCN synthesis.

### Protein interaction networks

**Figure 9:**
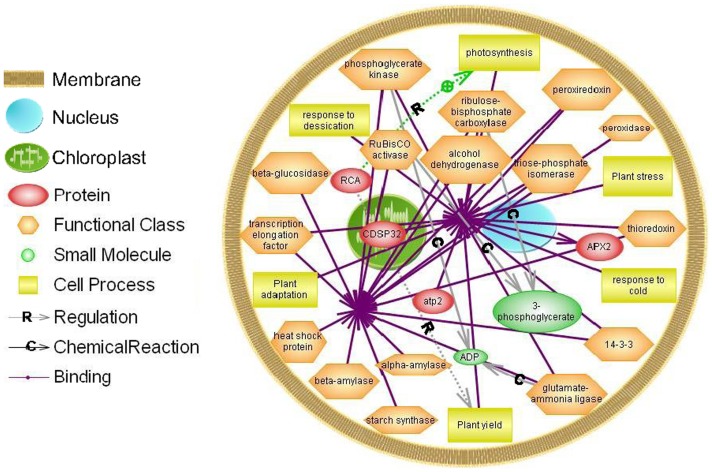
Biological networks generated for combination of twelve differential proteins. Fifteen differentially up-regulated proteins including ATP synthase subunit beta, alcohol dehydrogenase, beta-glucosidase, phosphoglycerate kinase, triose phosphate isomerase, RCA, Rubisco, APX2, CDSP3, peroxiredoxin, thioredoxin translation elongation factor, glutamate-ammonia ligase, chaperone and 14-3-3 in cassava autotetraploid genotypes were used to generate a protein-protein interaction network through Pathway Studio analysis. Regulation is marked as an arrow with R, Chemical Reaction as an arrow with C and Binding as an arrow without any marks. The entity table, relation table and reference table data were presented in in Tables S1, S2, S3.

All differential proteins identified in cassava autotetraploid genotypes were used to generate a wider protein interaction map by employing a Pathway Studio software program (Fig. 9). The relationships of binding and regulation were established for 15 differential proteins, responding to plant photosynthesis, yield, adaptation and stresses. CDSP32 is localized in the chloroplast. There are direct interactions between 15 up-regulated proteins, including ATP synthase subunit beta, alcohol dehydrogenase, beta-glucosidase, phosphoglycerate kinase, triose phosphate isomerase (TPI), Rubisco activase (RCA), Rubisco, APX2, CDSP3, peroxiredoxin (PrxQ), thioredoxin, translation elongation factor, glutamate-ammonia ligase, chaperone and 14-3-3 proteins, whereas RCA can establish relations with other proteins through regulating the processes of photosynthesis and plant yield (Tables S1, S2, S3).

## Discussion

Sheffield et al. (2006) compared proteomes between cassava fibrous and tuberous roots and found 292 differentially expressed spots on gels. Of those, 232 proteins were identified [6]. Li et al. (2010) identified 383 proteins from cassava somatic embryos, plantlets and tuberous roots using LC-ESI-MS/MS [3]. These data were helpful to understand proteome patterns between cassava different tissues. To further understand the mechanism of cassava whole-genome duplication induced by colchicine, we first investigated the significant changes in the proteome patterns of cassava diploid and autotetraploid genotypes and assess the potentially cassava-polyploid breeding. Differential protein spots that were found to contribute to this variation included proteins of eight functional categories (Table 1 and Fig. 4), in which there are direct interactions among 15 up-regulated proteins (Fig. 9). Furthermore, the expression levels of the differential proteins in cassava autotetraploid genotype may be directly associated with plant photosynthesis, yield, adaptation and stresses (Fig. 4 and Tables S1, S2, S3).

Effects of polyploid induction on the proteome were considerably pronounced. Statistical analysis showed clear differences in protein patterns between autotetraploid and diploid genotypes. In the present work, the proteins associated with plant defense system including defense proteins [beta-glucosidase (spots 9, 10), chloroplast latex aldolase-like protein (spot 24), metacaspase (spot 29), CDSP3 (spot 33), thioredoxin-like protein (spot 61)], detoxifying and antioxidant proteins were highly accumulated in cassava NZ199 autotetraploid genotype compared with diploid plants. Beta-glucosidase is a key enzyme regulating the abscisic acid (ABA) pool in plants under stresses [29], [30]. Chloroplast latex aldolase-like protein plays a role in defense and stress responses [31]. CDSP32 (a chloroplastic drought-induced stress protein) and thioredoxin-like protein, induced by environmental stress conditions are known to participate in the response to oxidative and drought stresses [32]–[34]. It is reported that plant metacaspases, a family of cysteine proteases structurally related to caspases, play important roles in biotic and abiotic stress-induced programmed cell death [35], [36]. PrxQ (spot 36) and APX (spots 39, 44, 45) are reported to participate in the protection against oxidative damage [25], [32]. Moreover, the evidences of salt stress on cassava diploid and autotetraploid genotypes showed that up-regulated proteins associated with plant defense could result in the increase of cassava plantlet tolerance to salt stress (Fig. 7 and Table 3). Interestingly, 14-3-3 protein (spot 37), a signal transformation- related protein, was up-regulated by more than 4.73-fold in autotetraploid genotype compared to diploid (Table 1). Previous studies revealed that multiple members of the 14-3-3 regulatory protein family act as mediators in ABA signaling through direct interaction with ABA-responsive element binding factors, and then alter the expression of related genes to enhance plant resistance against salinity or drought [37]. In addition, the previous studies described that the anatomical alterations of cassava polyploid plants, showing differences in density, compaction and thickness of parenchyma cells, may confer upon high tolerance to drought in tetraploid plant. The large number of vessel groupings in the tetraploid type may also maintain a larger quantity of water than in case of fewer ones in diploid plants [2], [38]. All above described results provide evidences at proteomic and anatomical levels to support polyploid genotype owning high tolerance to stresses.

Polyploids consistently exhibit larger mesophyll cells with more chloroplasts and greater photosynthetic capacities per cell than their diploid progenitors [39], [40]. The causes of these differences at the level of underlying proteins are unknown. In the present study cassava polyploid genotype could increase the expressed levels of 6 photosynthesis related proteins including nuclear encoded precursor to chloroplast protein (spot 4) [41], Rubisco large subunit-binding protein subunit beta (spot 8), oxygen evolving enhancer protein (spot 22) [25], RCA (spot 31), cytochrome b6-f complex iron-sulfur subunit (spot 55) [42] and rubisco (spot 60) (Table 1). These proteins are very likely involved in enhancement of photosynthesis, carbohydrate and energy metabolism in cassava leaves [3]. Rubisco serves as the main gateway for inorganic carbon to enter metabolic pathways in most ecosystems and hence is unique in its importance to support life. It catalyzes the key reaction in the photosynthetic assimilation of CO_2_ [43]. RCA could regulate the processes of plant photosynthesis and then affect the plant yield (Fig. 9). The evidences of photosynthetic activities in cassava polyploid plantlets showed that up-regulated proteins associated with photosynthesis could result in the increase of photosynthetic capacities in cassava autotetraploid genotype in comparison to diploid genotype (Fig. 6 and Table 2).

The up-regulated proteins involved in carbohydrate and energy metabolism including ATP synthase (spots 13–16, 30, 64, 65), phospoglycerate kinase (spot 26), alcohol dehydrogenase (ADH, spot 28) and TPI (spots 43, 46) (Table 1). Mitochondrial ATP synthase plays a role in mitochondrial changes through the life cell [44]. Phosphoglycerate kinase catalyzes the phosphorylation of 3-phosphoglycerate to 1, 3-diphosphoglycerate using ATP within the Calvin-Benson cycle [45]. ADH plays a significant role in sugar metabolism [46]. TPI catalyzes the reversible conversion of dihydroxyacetone phosphate to glyceraldehyde-3-phosphate and is involved in many pathways including glycolysis, Calvin cycle, and glycerol metabolism [15], [47]. However, we also detected 4 down-regulated spots including NAD(P)-binding Rossmann-fold-containing protein (spot 35) and ATP-binding protein (spots 47–49) in autotetraploid genotype. NAD(P)-binding Rossmann-fold-containing protein plays a role in protein folding [48]. ATP binding proteins, a large and important class, are kinases, which transfer the gamma phosphate from ATP to substrates; however, the functions of both proteins in cassava tetraploid genotype are unclear. The differential proteins regarding photosynthesis, carbohydrate and energy metabolism in autotetraploid genotype indicate that polyploidy would differ phenotypically from their diploid progenitors.

The previous studies showed that glutamate-ammonia ligase (GLUL or GS, previous name: glutamine synthetase) (spot 27), a key enzyme in the GS–glutamate synthase cycle, plays a pivotal role in the recycling of NH4^+^ that is released during photorespiration by generating glutamate from NH4^+^ and glutamine[49]. There are two isoforms of GS in higher plants, cytosolic GS1 and chloroplastic GS2, with recycling of photorespiratory NH4^+^ depending on GS2 [50]. The present study demonstrated that cytosolic GLUL at a higher expression in tetraploid genotype than diploid plants. Likewise, translation factor (spot 2) and elongation factor Tu (spots 3, 20), which are associated with the process of protein biosynthesis, showed higher expression levels in polyploid plants.

In the present study, cassava tetraploid genotype increased the expressed levels of HCN metabolism-related proteins including linamarase (spot 6) and acetone-cyanhydrin lyase (spot 32) (Table 1). Linamarase is an enzyme found in the cell walls of cassava. The major cyanogen in cassava is linamarin stored in the vacuole. Upon tissue disruption linamarin is deglycosylated bylinamarase, producing acetone cyanohydrin. Acetone cyanohydrin can spontaneously decompose at pH>5.0 or temperature >35°C, or is enzymatically broken down by acetone cyanohydrin lyase, which is hydroxynitrile lyase involved in the catabolism of cyanogenic glycosides [51], to produce acetone and free cyanide [52]. The evidences of HCN detection by HPLC showed that up-regulated proteins associated with HCN metabolism could result in the increase of HCN content in cassava autotetraploid genotype in comparison to diploid genotype (Fig. 8I–IV).

The autopolyploid genotypic variation is obvious as 65 proteins were detected showing significant differences in the present study. The proteomic study based on iTRAQ showed that the levels of protein divergence were relatively high between *Arabidopsis* autopolyploid and allopolyploid plants [22]. Proteomics is a powerful tool to characterize varieties of autopolyploids and allopolyploids [21]. Allopolyploidy can accelerate evolution through rapid and reproducible genomic changes in the first generation of nascent polyploids, including elimination of DNA sequences, gene silencing, alteration of cytosine methylation and activation of genes [53], and sporadic genomic changes that occur during the lifetime of a polyploidy species, which are not possible at the diploid level, such as diversification of homoeoalleles via mutations [54]. The phenomenon would provide an agronomic point of view; constructing allopolyploids could be more pertinent for crop improvement.

## Conclusions

The results suggest that 2-DE combined MS and bioinformatics techniques are a valid method to detect the changes of cassava polyploidy genotypes in proteome levels. The present study differs from the previous genomic-level studies of polyploidy in which investigate the differentially expressed proteins in relation with cassava autotetraploid genotype. The functional classification and expression levels of differential proteins showed that polyploid formation would be a complicated process of polyploidization, in which polyploidy provides a reservoir of duplicate genes as substrates for crop potential innovation. Following cassava polyploidization, new gene copies may undergo modifications allowing functional diversification in plants through the biological network of protein-protein interaction and the functional analyses of differential proteins. A useful proteome data set we provided would be helpful to predict the tolerance mechanism at a protein level of cassava polyploidy plants against environmental stresses. Further analysis of the diversity levels and the proteome patterns of duplicate gene not only traces variation of polyploids, but also provide insights into improvement of cassava polyploidy breeding in increasing photosynthesis and resistance efficiencies.

## Supporting Information

Figure S1 Scatter plot showed the ratios of the relative volumes. A, Uses spots for normalization, detected spots∼500, reproducible spots∼300; B, Scatter plots of reproducible protein spots on two 2-DE images from diploid and autotetraploid genotypes, respectively.
(DOC)

Figure S2 3-D maps of 65 differential proteins generated by Delta2D software based on the abundance of spots in 2-DE maps. a, differential spots from diploid genotype, b; differential spots from autotetraploid genotype. Blue circles indicated the location of protein spots from diploid genotype; yellow circles indicated spots from autotetraploid genotype. White letters indicated down-regulated; black letters indicated up-regulated. The numbering corresponded to the 2-DE gel in Fig. 2.
(DOC)

Table S1 Entity table views of protein-protein interactions in biological networks generated for cassava polyploid genotypes.
(DOC)

Table S2 Relation table views of protein-protein interactions in biological networks generated for cassava polyploid genotypes.
(DOC)

Table S3 Reference table views of protein-protein interactions in biological networks generated for cassava polyploid genotypes.
(DOC)
