# Supplementary figures and images for: Comparison of Leaf Proteomes of Cassava (Manihot esculenta Crantz) Cultivar NZ199 Diploid and Autotetraploid Genotypes
Source: PLoS One. 2014 Apr 11;9(4):e85991. doi: 10.1371/journal.pone.0085991 (PMC3984080; doi:10.1371/journal.pone.0085991)

**Figure S1**

**
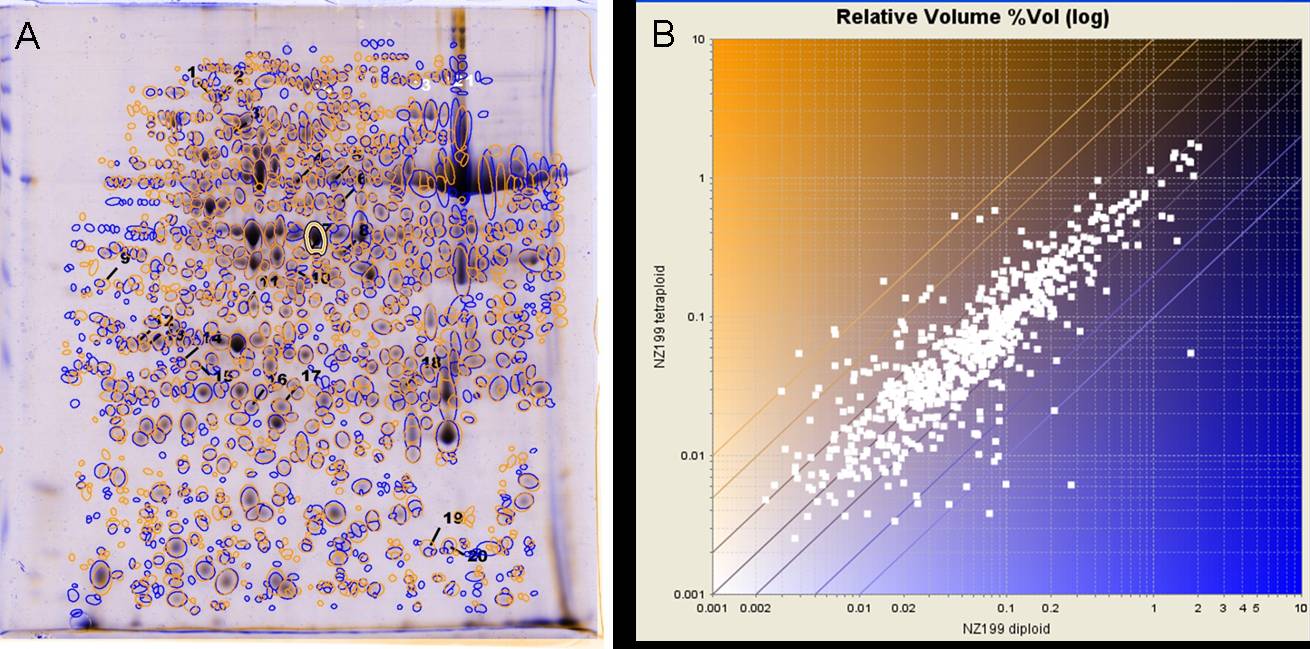
**

Supplement: Figure S1 — Scatter plot showed the ratios of the relative volumes. A, Uses spots for normalization, detected spots∼500, reproducible spots∼300; B, Scatter plots of reproducible protein spots on two 2-DE images from diploid and autotetraploid genotypes, respectively. (DOC) [file pone.0085991.s001.doc]

**Figure S2**

**
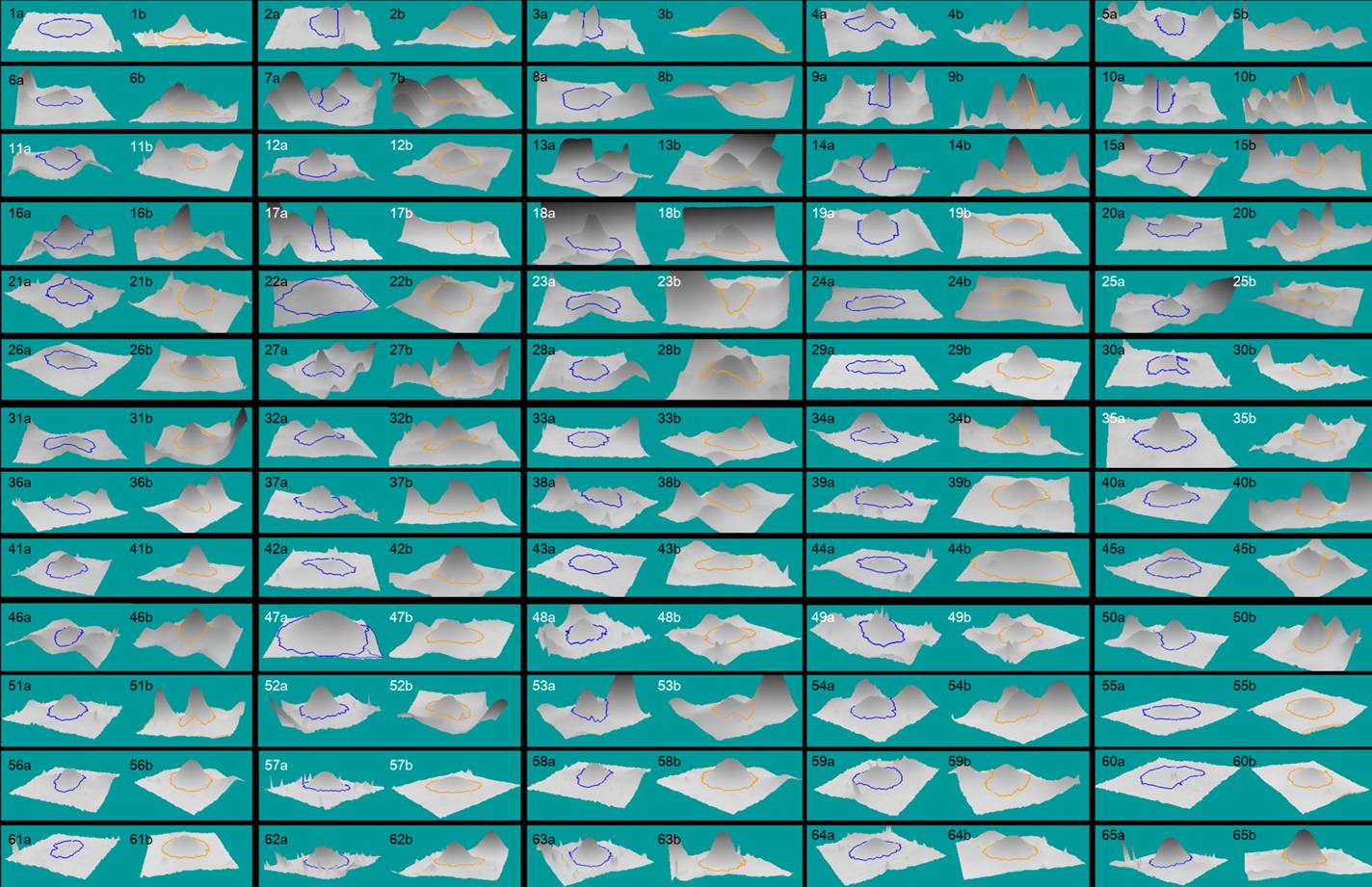
**

Supplement: Figure S2 — 3-D maps of 65 differential proteins generated by Delta2D software based on the abundance of spots in 2-DE maps. a, differential spots from diploid genotype, b; differential spots from autotetraploid genotype. Blue circles indicated the location of protein spots from diploid genotype; yellow circles indicated spots from autotetraploid genotype. White letters indicated down-regulated; black letters indicated up-regulated. The numbering corresponded to the 2-DE gel in Fig. 2. (DOC) [file pone.0085991.s002.doc]
