# Supplementary material for: Comparison of Leaf Proteomes of Cassava (Manihot esculenta Crantz) Cultivar NZ199 Diploid and Autotetraploid Genotypes
Source: PLoS One. 2014 Apr 11;9(4):e85991. doi: 10.1371/journal.pone.0085991 (PMC3984080; doi:10.1371/journal.pone.0085991)
Supplement: Table S1 — Entity table views of protein-protein interactions in biological networks generated for cassava polyploid genotypes. (DOC) [file pone.0085991.s003.doc]

**Table S1**

| Name | Type | Description | Connectivity | Local Connectivity | Indegree | Outdegree |
| --- | --- | --- | --- | --- | --- | --- |
| photosynthesis | Cell Process |  | 49 | 2 | 1 | 1 |
| Plant adaptation | Cell Process |  | 3 | 1 | 0 | 1 |
| Plant stress | Cell Process |  | 4 | 1 | 0 | 1 |
| Plant yield | Cell Process |  | 9 | 2 | 1 | 1 |
| response to cold | Cell Process |  | 2 | 1 | 0 | 1 |
| response to dessication | Cell Process |  | 3 | 1 | 0 | 1 |
| 14-3-3 | Functional Class |  | 4 | 2 | 0 | 2 |
| alcohol dehydrogenase | Functional Class |  | 5 | 1 | 0 | 1 |
| alpha-amylase | Functional Class |  | 3 | 1 | 0 | 1 |
| beta-amylase | Functional Class |  | 2 | 1 | 0 | 1 |
| beta-glucosidase | Functional Class |  | 2 | 1 | 0 | 1 |
| glutamate-ammonia ligase | Functional Class |  | 4 | 3 | 0 | 3 |
| heat shock protein | Functional Class |  | 1 | 1 | 0 | 1 |
| peroxidase | Functional Class |  | 1 | 1 | 0 | 1 |
| peroxiredoxin | Functional Class |  | 3 | 2 | 0 | 2 |
| phosphoglycerate kinase | Functional Class |  | 5 | 3 | 0 | 3 |
| ribulose-bisphosphate carboxylase | Functional Class |  | 4 | 3 | 0 | 3 |
| RuBisCO activase | Functional Class |  | 1 | 1 | 0 | 1 |
| starch synthase | Functional Class |  | 7 | 1 | 0 | 1 |
| thioredoxin | Functional Class |  | 4 | 2 | 0 | 2 |
| transcription elongation factor | Functional Class |  | 3 | 2 | 0 | 2 |
| triose-phosphate isomerase | Functional Class |  | 7 | 2 | 0 | 2 |
| APX2 | Protein | ascorbate peroxidase2 | 9 | 1 | 1 | 0 |
| atp2 | Protein | ATP synthase beta subunit | 2 | 1 | 0 | 1 |
| CDSP32 | Protein |  | 3 | 1 | 0 | 1 |
| RCA | Protein |  | 15 | 2 | 0 | 2 |
| 3-phosphoglycerate | Small Molecule |  | 16 | 2 | 2 | 0 |
| ADP | Small Molecule |  | 151 | 2 | 2 | 0 |
