# Supplementary material for: Comparison of Leaf Proteomes of Cassava (Manihot esculenta Crantz) Cultivar NZ199 Diploid and Autotetraploid Genotypes
Source: PLoS One. 2014 Apr 11;9(4):e85991. doi: 10.1371/journal.pone.0085991 (PMC3984080; doi:10.1371/journal.pone.0085991)
Supplement: Table S2 — Relation table views of protein-protein interactions in biological networks generated for cassava polyploid genotypes. (DOC) [file pone.0085991.s004.doc]

**Table S2**

| Relation | Type | Sentence | MedLine Reference | Connectivity | # of References |
| --- | --- | --- | --- | --- | --- |
| RCA --+> photosynthesis | Regulation | This indicated that virus infection caused the decreases of Rubisco and RCA in host plant, which then affected plant photosynthesis., Ribulose-1, 5-bisphosphate carboxylase/oxygenase activase (RCA) in the thylakoid membrane has been shown to play a role in protection and regulation of photosynthesis under moderate heat stress., To test the hypothesis that thermostable RCA can improve photosynthesis under elevated temperatures, we used gene shuffling technology to generate several Arabidopsis thaliana RCA1 (short isoform) variants exhibiting improved thermostability. | 15839204:5, 20478969:0, 17933901:2 | 2 | 3 |
| RCA ---> Plant yield | Regulation | Correlation of gene expression levels with three other traits indicates that RCA genes could play an important role in regulating soybean photosynthetic capacity and seed yield., The positive effects of shuffled thermostable RCA variants on ribulose-1,5-bisphosphate carboxylase/oxygenase activation state, rates of photosynthesis, and growth under moderate heat stress clearly demonstrate that RCA is a limiting factor in plant productivity under heat stress and provides a new strategy for improving crop yield under such stress conditions. | 20032079:1050, 17933901:1080 | 2 | 2 |
| ATP; ADP; phosphoglycerate kinase; 3-phosphoglycerate; 1,3-biphosphoglycerate | ChemicalReaction |  |  | 5 | 0 |
| Pi; glutamate-ammonia ligase; L-glutamine; ATP; L-glutamate; ADP; NH3 | ChemicalReaction |  |  | 7 | 0 |
| CO2; ribulose-bisphosphate carboxylase; Ribose-1,5-diphosphate; 3-phosphoglycerate; H2O; H+ | ChemicalReaction |  |  | 6 | 0 |
| Triose-phosphate isomerase; transcription elongation factor; CDSP32; Plant adaptation; response to dessication; Plant stress; Plant yield; response to cold; peroxiredoxin; 14-3-3; thioredoxin; glutamate-ammonia ligase; ribulose-bisphosphate carboxylase; atp2; APX2; photosynthesis; phosphoglycerate kinase | Binding |  |  | 17 | 0 |
| Phosphoglycerate kinase; Transcription elongation factor; beta-glucosidase; heat shock protein; alpha-amylase; beta-amylase; starch synthase; glutamate-ammonia ligase; 14-3-3; thioredoxin; peroxiredoxin; peroxidase; Triose-phosphate isomerase; alcohol dehydrogenase; ribulose-bisphosphate carboxylase; RuBisCO activase | Binding |  |  | 16 | 0 |
