# Supplementary material for: Comparison of Leaf Proteomes of Cassava (Manihot esculenta Crantz) Cultivar NZ199 Diploid and Autotetraploid Genotypes
Source: PLoS One. 2014 Apr 11;9(4):e85991. doi: 10.1371/journal.pone.0085991 (PMC3984080; doi:10.1371/journal.pone.0085991)
Supplement: Table S3 — Reference table views of protein-protein interactions in biological networks generated for cassava polyploid genotypes. (DOC) [file pone.0085991.s005.doc]

**Table S3**

| Relation | Status | Type | Sentence | MedLine Reference |
| --- | --- | --- | --- | --- |
| RCA --+> photosynthesis |  | Regulation | This indicated that virus infection caused the decreases of Rubisco and RCA in host plant, which then affected plant photosynthesis. | 15839204:5 |
| RCA --+> photosynthesis |  | Regulation | Ribulose-1, 5-bisphosphate carboxylase/oxygenase activase (RCA) in the thylakoid membrane has been shown to play a role in protection and regulation of photosynthesis under moderate heat stress. | 20478969:0 |
| RCA --+> photosynthesis |  | Regulation | To test the hypothesis that thermostable RCA can improve photosynthesis under elevated temperatures, we used gene shuffling technology to generate several Arabidopsis thaliana RCA1 (short isoform) variants exhibiting improved thermostability. | 17933901:2 |
| RCA ---> Plant yield |  | Regulation | Correlation of gene expression levels with three other traits indicates that RCA genes could play an important role in regulating soybean photosynthetic capacity and seed yield. | 20032079:1050 |
| RCA ---> Plant yield |  | Regulation | The positive effects of shuffled thermostable RCA variants on ribulose-1,5-bisphosphate carboxylase/oxygenase activation state, rates of photosynthesis, and growth under moderate heat stress clearly demonstrate that RCA is a limiting factor in plant productivity under heat stress and provides a new strategy for improving crop yield under such stress conditions. | 17933901:1080 |
